# cGMP Imaging in Brain Slices Reveals Brain Region-Specific Activity of NO-Sensitive Guanylyl Cyclases (NO-GCs) and NO-GC Stimulators

**DOI:** 10.3390/ijms19082313

**Published:** 2018-08-07

**Authors:** Stefanie Peters, Michael Paolillo, Evanthia Mergia, Doris Koesling, Lea Kennel, Achim Schmidtko, Michael Russwurm, Robert Feil

**Affiliations:** 1 Interfakultäres Institut für Biochemie, University of Tübingen, 72076 Tübingen, Germany; stefanie.peters07@gmail.com (S.P.); michaelpaolillo1@gmail.com (M.P.); 2 Institut für Pharmakologie und Toxikologie, Ruhr-Universität Bochum, 44801 Bochum, Germany; mergia@evanthia.de (E.M.); doris.koesling@ruhr-uni-bochum.de (D.K.); michael.russwurm@ruhr-uni-bochum.de (M.R.); 3 Pharmakologisches Institut für Naturwissenschaftler, University of Frankfurt, 60438 Frankfurt am Main, Germany; kennel@em.uni-frankfurt.de (L.K.); schmidtko@em.uni-frankfurt.de (A.S.)

**Keywords:** Cyclic GMP, nitric oxide, guanylyl cyclase, NO-GC stimulators, Purkinje cells, cerebellar granule cells, striatum, hippocampal neurons, FRET imaging, transgenic mice

## Abstract

Impaired NO-cGMP signaling has been linked to several neurological disorders. NO-sensitive guanylyl cyclase (NO-GC), of which two isoforms—NO-GC1 and NO-GC2—are known, represents a promising drug target to increase cGMP in the brain. Drug-like small molecules have been discovered that work synergistically with NO to stimulate NO-GC activity. However, the effects of NO-GC stimulators in the brain are not well understood. In the present study, we used Förster/fluorescence resonance energy transfer (FRET)-based real-time imaging of cGMP in acute brain slices and primary neurons of cGMP sensor mice to comparatively assess the activity of two structurally different NO-GC stimulators, IWP-051 and BAY 41-2272, in the cerebellum, striatum and hippocampus. BAY 41-2272 potentiated an elevation of cGMP induced by the NO donor DEA/NO in all tested brain regions. Interestingly, IWP-051 potentiated DEA/NO-induced cGMP increases in the cerebellum and striatum, but not in the hippocampal CA1 area or primary hippocampal neurons. The brain-region-selective activity of IWP-051 suggested that it might act in a NO-GC isoform-selective manner. Results of mRNA in situ hybridization indicated that the cerebellum and striatum express NO-GC1 and NO-GC2, while the hippocampal CA1 area expresses mainly NO-GC2. IWP-051-potentiated DEA/NO-induced cGMP signals in the striatum of NO-GC2 knockout mice but was ineffective in the striatum of NO-GC1 knockout mice. These results indicate that IWP-051 preferentially stimulates NO-GC1 signaling in brain slices. Interestingly, no evidence for an isoform-specific effect of IWP-051 was observed when the cGMP-forming activity of whole brain homogenates was measured. This apparent discrepancy suggests that the method and conditions of cGMP measurement can influence results with NO-GC stimulators. Nevertheless, it is clear that NO-GC stimulators enhance cGMP signaling in the brain and should be further developed for the treatment of neurological diseases.

## 1. Introduction

The second messenger cyclic 3’,5’-guanosine monophosphate (cGMP) regulates various physiological processes such as cellular growth and contractility, cardiovascular homeostasis, inflammation, sensory transduction, and neuronal plasticity and learning [1,2]. cGMP is generated from GTP by either nitric oxide (NO)-sensitive guanylyl cyclases (NO-GCs) [3,4] or membrane-bound particulate guanylyl cyclases that are activated by peptides like the natriuretic peptides, guanylin/uroguanylin, or enterotoxins [5]. The mammalian NO-GC is a heterodimeric hemoprotein that consists of a larger α subunit and a smaller β subunit. Currently, two NO-GC isoforms are known: α_1_β_1_ (termed NO-GC1) and α_2_β_1_ (termed NO-GC2) [6]. The prosthetic heme group of NO-GC is bound to the β_1_ subunit through the axial ligand histidine 105 [7] and the heme-binding motif tyrosine 135 and arginine 139 [8]. NO binding to the heme Fe^2+^ leads to the formation of nitrosyl heme [9]. This disrupts the histidine 105-iron bond and is required but not sufficient for activation of the enzyme [7,10,11,12]. Once generated, cGMP acts on several downstream effectors including cyclic nucleotide-gated ion channels, cGMP-dependent protein kinases, and phosphodiesterases (PDEs) that degrade cAMP and/or cGMP [2].

NO-GC represents a promising therapeutic target. Since reduced levels of cGMP signaling have been associated with several cardiovascular and neurological disorders, drugs are being developed that enhance NO-GC activity and lead to an increased cellular cGMP concentration [13]. However, it has been reported that cardiovascular diseases are associated with resistance to treatment with NO-releasing compounds such as organic nitrates [14]. Small-molecule NO-GC “stimulators” have been discovered that are structurally not related to NO [15] and thought to sensitize NO-GC to low levels of bioavailable NO by stabilizing the nitrosyl-heme complex thus maintaining the enzyme in its active configuration [16]. They are considered to enhance NO-GC activity in synergy with NO. The NO-GC stimulator Bay 41-2272 is a pyrazole-pyridine derivative developed by Bayer AG [17,18]. It acts in a heme-dependent manner since it does not stimulate a heme-free variant of NO-GC [19]. However, it is interesting to note that Bay 41-2272 may increase cGMP concentrations not only by sensitizing NO-GC, but also by inhibiting the cGMP-degrading PDE5 [20,21]. Impaired NO-cGMP signaling is associated with arterial hypertension [22,23]. It has been shown that NO-GC stimulators like Bay 41-2272 and YC-1 significantly reduce mean arterial pressure in hypertensive rats [22]. Furthermore, inhaled Bay 41-2272 induced selective pulmonary vasodilation in ovine and rat models of pulmonary hypertension [24]. Stasch and colleagues developed riociguat (Adempas^®^), the first potent, orally available NO-GC stimulator that was approved to treat patients with pulmonary hypertension [25,26,27]. IWP-051, developed by Ironwood Pharmaceuticals, is a novel orally bioavailable NO-GC stimulator that utilizes a biaryl pyrazole structure instead of the fused ring structure of the above mentioned Bayer compounds [28]. It stimulates NO-GC heme-dependently and in synergy with NO. In normotensive rats, administration of IWP-051 (ranging from 1 to 100 mg/kg) decreased mean arterial pressure in a dose-dependent manner [28]. NO-GC stimulators are promising drugs for the treatment of cardiovascular diseases, but their development for the treatment of disorders of the brain is lagging behind, at least in part, because the effects of these drugs on the nervous system are not well understood and they have to cross the blood brain barrier. cGMP-elevating drugs acting on the brain are, however, of enormous interest, since cGMP plays a crucial role in physiological and pathophysiological processes of the nervous system including synaptic plasticity, cognition, and neurodegeneration [29,30].

In 2013, Thunemann et al. [31] generated a mouse line that expresses the Förster/fluorescence resonance energy transfer (FRET)-based cGMP indicator cGi500 globally in all tissues and cells. This mouse line is named R26-CAG-cGi500(L1). cGi500 consists of the tandem cGMP-binding sites of the bovine cGMP-dependent protein kinase type I flanked by cyan fluorescent protein (CFP) and yellow fluorescent protein (YFP). Its sensitivity for cGMP (EC_50_) was reported to be 500 nM [32]. In the absence of cGMP, the fluorophores are in close proximity and after excitation of CFP, FRET occurs from CFP (FRET donor) to YFP (FRET acceptor) resulting in fluorescence emission from the latter. Upon an increase of the cGMP concentration and binding of cGMP to the sensor, it undergoes a conformational change that results in an increased distance between CFP and YFP and a lower FRET efficiency. This leads to an increased emission of CFP at 480 nm and a decreased emission of YFP at 535 nm. The ratio of CFP/YFP emission can directly be correlated to the cGMP concentration [32]. With the availability of the cGMP sensor mouse, real-time monitoring of cGMP is no longer restricted to transfected cells in culture, but also feasible in living tissues and animals. Studies with cGMP sensor mice showed, for instance, that different types of smooth muscle cells have different sensitivities in regard to cGMP production stimulated by the NO donor 2-(N,N-diethylamino)-diazenolate-2-oxide diethylammonium salt (DEA/NO), atrial natriuretic peptide (ANP) and C-type natriuretic peptide (CNP). Moreover, robust NO-induced cGMP signals could be recorded in blood vessels of isolated retina and in the cremaster microcirculation of anesthetized mice [31,33,34].

A related cGMP sensor mouse line, named R26-CAG-cGi500(L2), carries a loxP-flanked stop cassette preceding the cGi500-encoding sequence and enabling Cre-activatable expression of cGi500 [31]. The stop cassette encodes membrane-targeted tandem dimer tomato (mT). Thus, this transgene expresses mT and cGi500 before and after Cre recombination, respectively. Depending on where Cre recombinase is expressed, tissue/cell type-specific expression of cGi500 can be achieved [31]. In the present study, we used acute brain slices of neuron-specific cGMP sensor mice for visualization and comparative characterization of cGMP signals evoked by the NO-GC stimulators Bay 41-2272 and IWP-051, with a focus on the effects of IWP-051 on the cerebellum, striatum and hippocampus. While both IWP-051 and Bay 41-2272 potentiated NO-induced cGMP signals in neurons, we also observed interesting differences with respect to their activity in different brain regions. These findings indicate that it might be reasonable to develop NO-GC stimulators for the treatment of central nervous system (CNS) diseases.

## 2. Results

### 2.1. IWP-051 Potentiates NO-Induced cGMP Signals in Purkinje Cells and Granule Neurons of the Cerebellum as Well as in the Striatum

**Figure 1:**
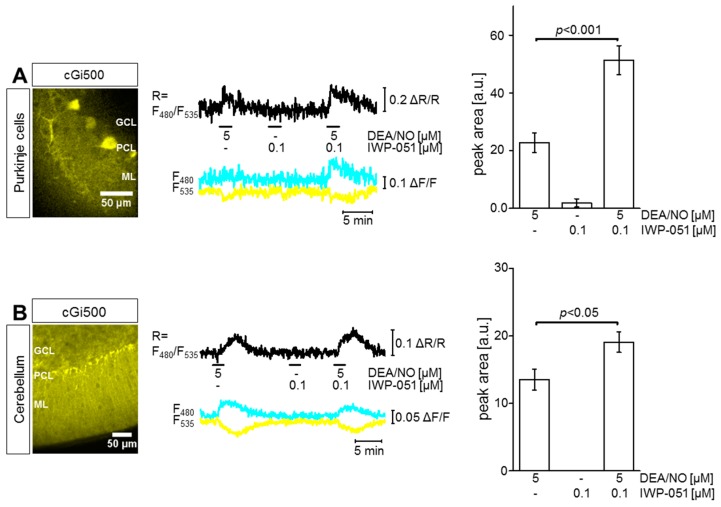
IWP-051 potentiates 2-(*N*,*N*-diethylamino)-diazenolate-2-oxide diethylammonium salt (DEA/NO)-induced cGMP signals in Purkinje cells and in the granule cell layer of the cerebellum. (**A**) Förster/fluorescence resonance energy transfer (FRET)-based cGMP imaging was performed in acute cerebellar brain slices from L7Cre;R26-CAG-cGi500(L2) mice with Purkinje cell-specific expression of cGi500. Yellow color represents the YFP fluorescence of cGi500 in Purkinje cells (left). A representative measurement of a Purkinje cell is shown (middle). During measurement, DEA/NO, IWP-051 or a combination of both was applied in the indicated concentrations (middle, black horizontal bars). Black, cyan and yellow traces represent cyan fluorescent protein (CFP)/yellow fluorescent protein (YFP) ratio, CFP trace and YFP trace, respectively. Statistical analysis (right) was performed with peak areas of respective cGMP signals; data are shown as mean ± SEM (*n* = 5 ROIs from one brain slice); (**B**) FRET-based cGMP imaging was performed in acute cerebellar brain slices from NesCre;R26-CAG-cGi500(L2) mice expressing cGi500 in neurons and glia cells. Yellow color represents the YFP fluorescence of cGi500 in the cerebellum (left). A representative measurement of the granule cell layer (GCL) is shown (middle). Statistical analysis (right) was performed with peak areas of respective cGMP signals; data are shown as mean ± SEM (*n* = 9 ROIs from three brain slices). ML, molecular layer; PCL, Purkinje cell layer.

To visualize cGMP in real time under close-to-native conditions, acute brain slices were analyzed by FRET-based cGMP imaging with a spinning disk microscope. Slices were carbogen-gassed during preparation and measurements to keep the tissue alive. To investigate the effect of IWP-051 specifically in Purkinje cells of the cerebellum, the L7Cre;R26-CAG-cGi500(L2) mouse line was used. In these mice, cGi500 is specifically expressed in cerebellar Purkinje cells (Figure 1A left, yellow). The FRET graph and the statistical summary (Figure 1A middle and right, respectively) show that the application of DEA/NO (5 µM) led to a clear elevation of cGMP in Purkinje cells, confirming the presence of the NO-cGMP signaling cascade in this cell type. Application of the NO-GC stimulator IWP-051 (0.1 µM) alone did not affect the cGMP level in Purkinje cells, while the combination of IWP-051 with DEA/NO significantly potentiated the DEA/NO-induced cGMP level ~2.5-fold. This experiment demonstrated the potential of IWP-051 to stimulate NO-GC in Purkinje cells of the cerebellum.

**Figure 2:**
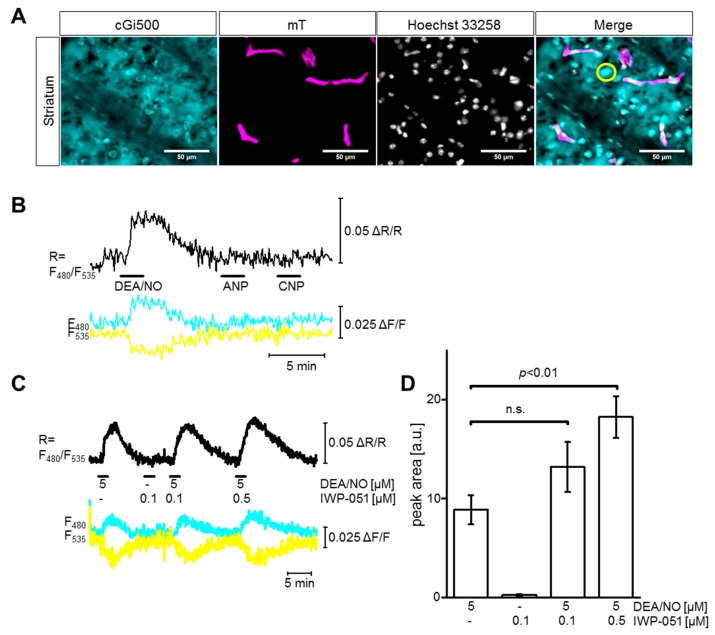
IWP-051 potentiates DEA/NO-induced cGMP signals in the striatum. (**A**) Representative pictures of the striatum in fixed NesCre;R26-CAG-cGi500(L2) brain sections showing expression of cGi500 (cyan) and membrane-targeted tomato protein (mT, magenta), staining of nuclei with Hoechst (grey), and an overlay of all three channels (Merge). The yellow circle shows a representative ROI chosen for analysis; (**B**) Representative cGMP measurement in an acute striatal brain slice from a NesCre;R26-CAG-cGi500(L2) mouse. During measurement, 5 µM DEA/NO, 0.25 µM ANP and 0.25 µM CNP were applied (black horizontal bars). Black, cyan and yellow traces represent CFP/YFP ratio, CFP and YFP, respectively; (**C**) Representative cGMP measurement in the striatum with DEA/NO and IWP-051 in the indicated concentrations (black horizontal lines). For cGMP imaging, striatal cells were selected that were separated from surrounding blood vessels to avoid false-positive signals (e.g., yellow circle in panel (**A**)); (**D**) Statistical analysis was performed with peak areas of the respective cGMP signals; data are shown as mean ± SEM (*n* = 6 ROIs from three brain slices); n.s., not significant. Similar results were obtained in independent experiments with additional mice and brain slices (see Figure 6A,C, wildtype).

**Figure 3:**
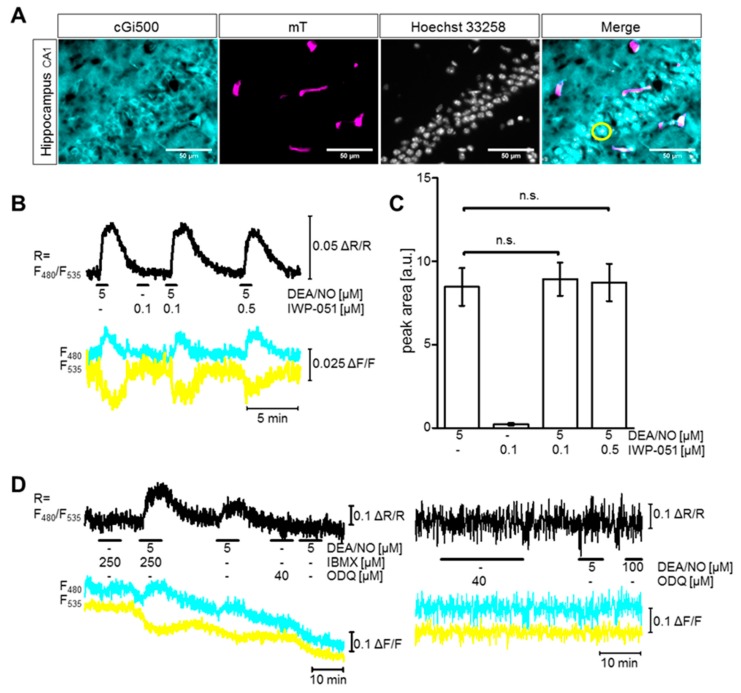
IWP-051 does not potentiate DEA/NO-induced cGMP signals in the hippocampal CA1 area. (**A**) Representative pictures of the hippocampal CA1 area in fixed NesCre;R26-CAG-cGi500(L2) brain sections showing expression of cGi500 (cyan) and membrane-targeted tomato protein (mT, magenta), staining of nuclei with Hoechst (grey), and an overlay of all three channels (Merge). The yellow circle shows a representative ROI chosen for analysis; (**B**–**D**) cGMP imaging of hippocampal cells in the CA1 region was performed in acute hippocampal brain slices from NesCre;R26-CAG-cGi500(L2) mice. To avoid false-positive signals, only regions of interest that were separated from surrounding blood vessels were analyzed (e.g., yellow circle in panel (**A**)). Black, cyan and yellow traces represent CFP/YFP ratio, CFP and YFP, respectively; (**B**) Representative experiment in which DEA/NO and IWP-051 were applied in the indicated concentrations (black horizontal lines) and (**C**) statistical analysis of peak areas of respective cGMP signals; data are shown as mean ± SEM (*n* = 9 ROIs from three brain slices); n.s., not significant. Similar results were obtained in independent experiments with additional mice and brain slices (see Figure 6B,D, wildtype). (**D**) Representative experiments in which 3-isobutyl-1-methylxanthin (IBMX) and 1H-[1,2,4]oxadiazolo[4 ,3-a]quinoxalin-1-one (ODQ) were applied before or in combination with DEA/NO application. The respective concentrations are indicated in the graphs. Similar results were obtained in at least three ROIs measured on two brain slices (IBMX and ODQ, left) or one brain slice (ODQ, right).

Figure 1B summarizes cGMP measurements performed in the granule cell layer (GCL) of acute cerebellar slices prepared from NesCre;R26-CAG-cGi500(L2) mice. These mice express cGi500 broadly in neural tissue including neurons and glial cells (Figure 1B left, yellow). It is important to note that these mice do not express the sensor in cerebral blood vessels (red structures in Figure 2A and Figure 3A). It is well known that blood vessels generate strong NO-induced cGMP signals. If they would express cGi500, then they would likely confound cGMP imaging of neural tissue. Similar to the cGMP signaling pattern observed in Purkinje cells, the application of DEA/NO (5 µM) resulted in a robust elevation of cGMP in the GCL, while IWP-051 (0.1 µM) alone was not effective. However, the combination of DEA/NO with IWP-051 significantly potentiated the magnitude of the cGMP signal in the GCL ~1.5-fold as compared to the signal induced by DEA/NO alone (Figure 1B middle and right). A similar potentiation of NO-induced cGMP signals by IWP-051 was detected in the Purkinje cell layer and granule cell layer of R26-CAG-cGi500(L1) mice with global expression of the cGMP sensor (data not shown).

Next, the NesCre;R26-CAG-cGi500(L2) mouse line was used to investigate the potential of IWP--051 to enhance NO-induced cGMP signals in the striatum. Figure 2A shows the expression of cGi500 in neural cells of the striatum (cyan). The fluorescence of mT (magenta) in small blood vessels of the striatum showed that these vessels did not express the cGMP indicator. An overlay of both channels with nuclear Hoechst staining (grey) demonstrated a high density of neural cells in the striatal area of interest (Merge). These cells were selected for FRET-based cGMP imaging experiments in acute brain slices. As documented in Figure 2B, DEA/NO (5 µM) induced a strong cGMP elevation in striatal cells, while the natriuretic peptides ANP (0.25 µM) and CNP (0.25 µM) were not effective. These results are in line with the literature describing the presence of the NO-cGMP pathway in the striatum [35]. In contrast to DEA/NO (5 µM), IWP-051 (0.1 µM) alone did not induce a detectable cGMP signal in the striatum. However, the combination of DEA/NO (5 µM) and IWP-051 (0.1 µM or 0.5 µM) led to increased peak areas of the NO-induced cGMP signals as compared to the application of DEA/NO alone (Figure 2C,D), with 0.5 µM IWP-051 resulting in a statistically significant ~2-fold potentiation (Figure 2D).

Together, these data showed that IWP-051 is a potent NO-GC stimulator that enhances NO-induced cGMP signals in Purkinje cells and granule neurons of the cerebellum as well as in neurons of the striatum.

### 2.2. IWP-051 Has No Detectable Effect on NO-Induced cGMP Signals in the Hippocampal CA1 Area of Acute Brain Slices

The cGi500 sensor was also expressed in the hippocampal CA1 area of NesCre;R26-CAG-cGi500(L2) mice (Figure 3A, cyan). Further analysis showed that, similar to the brain regions analyzed before, small blood vessels (magenta) were devoid of cGi500 expression. By performing nuclear co-staining with Hoechst dye (grey), the pyramidal cell layer in the hippocampal CA1 area could be visualized (Merge). This region was chosen for cGMP imaging in acute hippocampal brain slices. The application of DEA/NO (5 µM) triggered cGMP signals, but these signals were not increased by co-application of IWP-51 (0.1 µM or 0.5 µM) (Figure 3B,C). Thus, in contrast to the cerebellum and striatum, IWP-051 did not potentiate NO-induced cGMP signals in the hippocampal CA1 region.

IWP-051 could have been ineffective because the application of 5 µM DEA/NO had already increased the cGMP concentration to the maximum or to a level at which the cGMP sensor was already saturated. To exclude these possibilities, we performed control experiments with the nonspecific PDE inhibitor 3-isobutyl-1-methylxanthin (IBMX). Co-application of IBMX (250 µM) strongly increased cGMP elevation elicited by 5 µM DEA/NO (Figure 3D left), thus, refuting the possibility that application of NO alone had already resulted in a maximal cGMP response or saturation of the cGMP sensor. Furthermore, the baseline FRET/cGMP signal was not altered by application of the NO-GC inhibitor ODQ or of the PDE inhibitor IBMX (Figure 3D left and right) indicating that basal cGMP production via NO-GC as well as cGMP degradation via PDEs was very low to absent in our acute hippocampal brain slices. The functionality of ODQ as a NO-GC inhibitor was verified by the fact that subsequent application of DEA/NO did not elevate cGMP (Figure 3D left and right).

### 2.3. IWP-051 Potentiates NO-Induced cGMP Signals in Primary Cerebellar Granule Neurons, But Not in Primary Hippocampal Neurons

**Figure 4:**
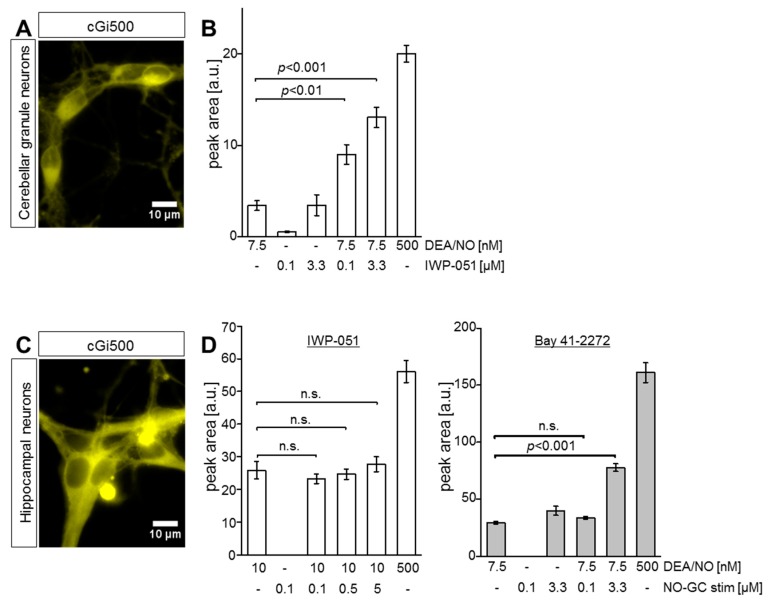
IWP-051 potentiates DEA/NO-induced cGMP signals in primary cerebellar granule neurons (CGNs), but not in hippocampal neurons (HNs). (**A**) Representative picture of primary cGi500-expressing CGNs (yellow) isolated from R26-CAG-cGi500(L1) mice; (**B**) Statistical analysis of cGMP imaging in CGNs, showing peak areas of cGMP signals evoked by DEA/NO and IWP-051. Respective concentrations are indicated below the graph; data are shown as mean ± SEM (*n* = 7 cells from two coverslips); (**C**) Representative picture of primary cGi500-expressing HNs (yellow) isolated from R26-CAG-cGi500(L1) mice; (**D**) Statistical analysis of cGMP imaging in HNs, showing peak areas of cGMP signals induced by DEA/NO and IWP-051 (left, *n* = 12 cells from two coverslips) or DEA/NO and Bay 41-2272 (right, *n* = 6 cells from one coverslip). Data are shown as mean ± SEM. Respective concentrations are indicated below the graphs; n.s. not significant.

To support the results of cGMP imaging in acute brains, we analyzed primary cultures of cerebellar granule neurons (CGNs) and hippocampal neurons (HNs) obtained from R26-CAG-cGi500(L1) mice. After confirming cGi500 expression in our primary CGNs and HNs (Figure 4A,C, respectively), FRET-based cGMP imaging was performed and peak areas of respective cGMP signals were evaluated. In line with the cGMP imaging data from acute brain slices, co-application of IWP-051 strongly potentiated NO-induced cGMP elevations in primary CGNs (Figure 4B), but not in primary HNs (Figure 4D left). In contrast to IWP-051, Bay 41-2272 had a clear potentiating effect on the NO-induced cGMP signal in HNs (Figure 4D right). These findings indicated potential differences in the activity profiles of the NO-GC stimulators IWP-051 and Bay 41-2272 in different brain regions. The application of a high DEA/NO concentration (500 nM) at the end of each experiment (Figure 4B,D) confirmed that the FRET/cGMP signals recorded in response to experimental concentrations of DEA/NO (7.5 nM or 10 nM) were not already maximal. This control also excludes sensor saturation as a cause for the failure to detect potentiation of NO-induced cGMP signals by IWP-051 in HNs.

Based on the data obtained by cGMP imaging of acute brain slices and isolated primary neurons, it appears that IWP-051 is a potent NO-GC stimulator in specific brain regions such as cerebellum and striatum but not in hippocampus, although the latter brain region can clearly produce NO-induced cGMP signals.

### 2.4. Expression Data and cGMP Imaging in Knockout Models Indicate a NO-GC1-Specific Activity of IWP-051

**Figure 5:**
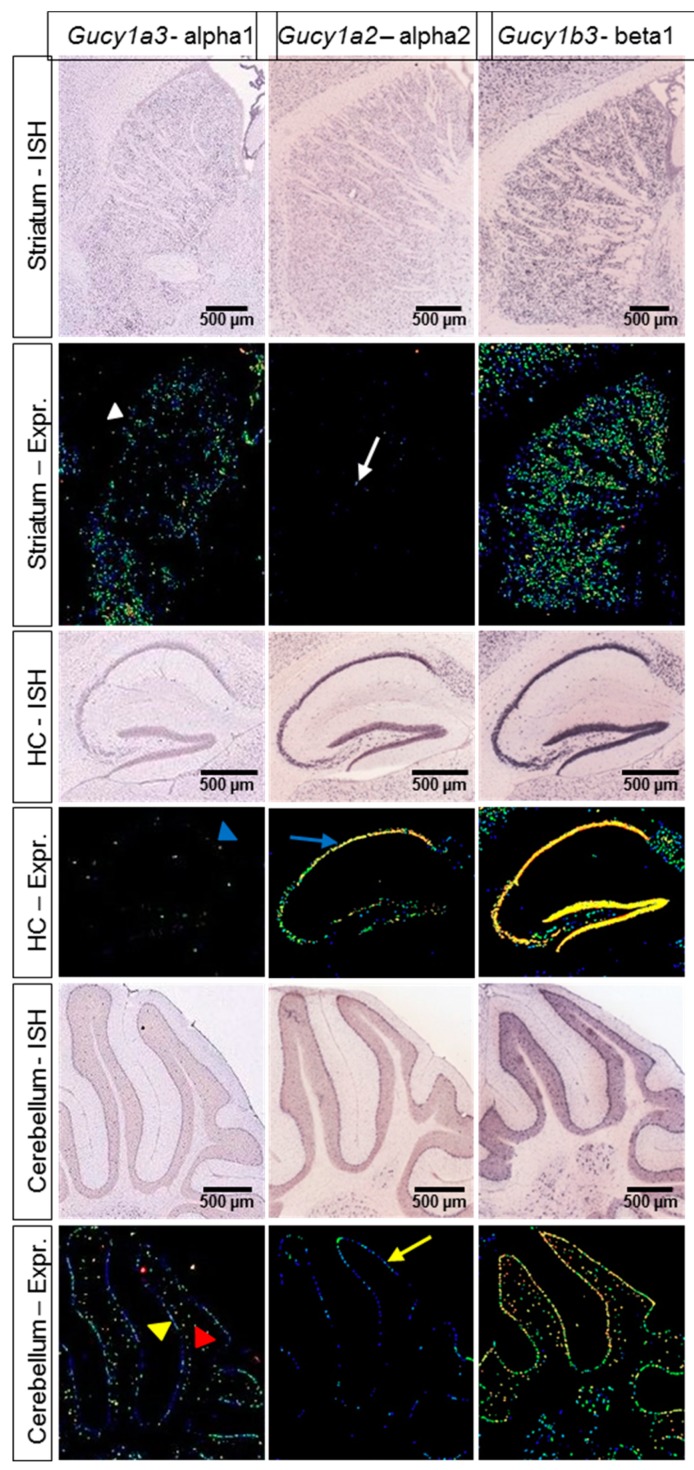
Expression of NO-GC1 mRNA is prominent in striatum and cerebellum, while NO-GC2 mRNA is strongly expressed in the hippocampus. Representative pictures of in situ hybridization (ISH) experiments showing mRNA expression of NO-GC subunits in striatum, hippocampus (HC) and cerebellum of an adult C57BL/6 mouse. Pictures labeled with “ISH” represent the original ISH staining, while pictures labeled with “Expr.” represent a heat map representing the relative amount of the respective mRNA (blue dots indicate low expression, yellow dots indicate high expression). NO-GC1 expression was detected in striatum (white arrowhead) and in the Purkinje cell layer (yellow arrowhead) and granule cell layer (red arrowhead) of the cerebellum. In the hippocampus, NO-GC1 expression was very low to undetectable (blue arrowhead), while NO-GC2 expression was high (blue arrow). Weak NO-GC2 expression was observed in the striatum (white arrow) and Purkinje cell layer of the cerebellum (yellow arrow). Data is taken from the Allen mouse brain atlas [36]. Image credit: Allen Institute.

The apparent brain region-specific activity profile of IWP-051 could be explained by a NO-GC isoform-specific activity of the compound. According to mRNA in situ hybridization data obtained by us (data not shown) or published in the Allen mouse brain atlas [36] (Figure 5), NO-GC1 is mainly expressed in striatum (white arrowhead), cerebellar Purkinje cells (yellow arrowhead), and cerebellar granule neurons (red arrowhead), but not in the hippocampus (blue arrowhead). In contrast, only few neurons in the striatum (white arrow) and cerebellum, mainly Purkinje cells (yellow arrow), show NO-GC2 expression, while the hippocampal dentate gyrus and CA1-CA3 area express high levels of NO-GC2 (blue arrow). A similar expression profile of the NO-GC isoforms was found in the rat brain [37], and mRNA analysis by real-time polymerase chain reaction showed that mouse hippocampus expresses a 3-fold higher mRNA level of NO-GC2 vs. NO-GC1 [38]. The activity profile of IWP-051 and the expression pattern of NO-GC1 and NO-GC2 in the striatum, cerebellum and hippocampus is consistent with isoform-specific potentiation of NO-GC1 but not NO-GC2 by IWP-051.

**Figure 6:**
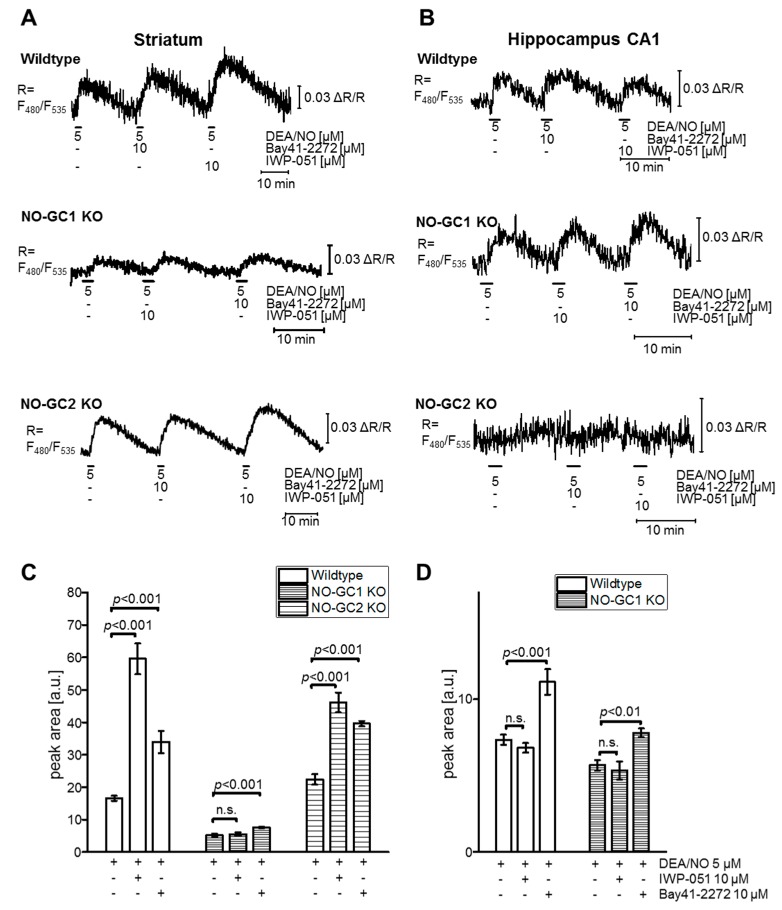
cGMP imaging in acute brain slices of NO-GC1 and NO-GC2 knockout mice reveals different activities of IWP-051 and Bay 41-2272 in (**A**,**C**) striatum and (**B**,**D**) hippocampus. Representative cGMP measurements (showing CFP/YFP ratios) performed (**A**) in the striatum and (**B**) hippocampus of wildtype, NO-GC1 KO and NO-GC2 KO mice that had been crossed to NesCre;R26-CAG-cGi500(L2) mice and, thus, expressed cGi500 in neurons and glia cells. DEA/NO, IWP-051, and Bay 41-2272 were applied in the indicated concentrations (black horizontal lines). Statistical analysis of respective cGMP signals in (**C**) striatum (wildtype, *n* = 22; NO-GC1 KO, *n* = 16; NO-GC2 KO, *n* = 16) and (**D**) hippocampus (wildtype, *n* = 22; NO-GC1 KO, *n* = 9) was performed with peak areas; data are shown as mean ± SEM (*n*-numbers represent number of evaluated ROIs from at least three brain slices per genotype); n.s., not significant. To avoid false-positive signals, only ROIs that were separated from surrounding blood vessels were analyzed. Note that the absence of detectable cGMP signals in the hippocampus of NO-GC2 KO precluded statistical analysis. For each genotype and brain region, similar results were obtained in independent experiments with brain slices from three mice.

To further substantiate the hypothesis that IWP-051 acts in the brain as NO-GC1-specific stimulator, while Bay 41-2272 stimulates both NO-GC1 and NO-GC2, FRET-based cGMP imaging was performed with acute brain slices of NesCre;R26-CAG-cGi500(L2) mice that expressed wildtype levels of NO-GC isoforms or were genetically deficient for NO-GC1 (NO-GC1 KO) or NO-GC2 (NO-GC2 KO) (Figure 6). Pilot experiments revealed that a concentration of 10 µM Bay 41-2272 was required to reliably detect a potentiating effect of this compound on DEA/NO-induced cGMP signals in brain slices. Thus, for comparative experiments IWP-051 and Bay 41-2272 were used in a concentration of 10 µM. As expected, in wildtype mice IWP-051 potentiated NO-induced cGMP levels in striatum but not hippocampus, while Bay 41-2272 was active in both brain regions (Figure 6A,B upper; Figure 6C,D). IWP-051 did not potentiate NO-induced cGMP signals in the striatum of NO-GC1 KO mice, while it was active in the striatum of NO-GC2 KO mice (Figure 6A middle and lower; Figure 6C). This analysis of knockout mice clearly showed that NO-GC1 but not NO-GC2 is required for the potentiating activity of IWP-051 in the striatum. In contrast to IWP-051, Bay 41-2272 enhanced NO-induced cGMP signals in the striatum of both NO-GC1 and NO-GC2 KO mice (Figure 6A middle and lower; Figure 6C), indicating that this NO-GC stimulator acts on both isoforms. The NO-induced cGMP signals in the striatum of NO-GC1 KO mice were smaller than in wildtype striatum, but could still be significantly potentiated by Bay 41-2272 (Figure 6C), presumably because the striatum expresses, in addition to NO-GC1, also a low level of NO-GC2 (Figure 5). Note that we did not detect NO-induced cGMP signals in the hippocampus of NO-GC2 KO mice (Figure 6B lower) precluding experiments with NO-GC stimulators in hippocampal brain slices from these mice.

Taken together, cGMP imaging in acute brain slices, in particular the comparative testing of IWP-051 and Bay 41-2272 in wildtype, NO-GC1 KO and NO-GC2 KO mice, and the expression profile of NO-GC isoforms, provided strong evidence for the notion that the brain-region-specific activity of IWP-051 was related to isoform-specific stimulation of NO-GC1 but not NO-GC2.

### 2.5. In Vitro Analysis of Brain Homogenates Does Not Show Isoform-Specific Activity of IWP-051

**Figure 7:**
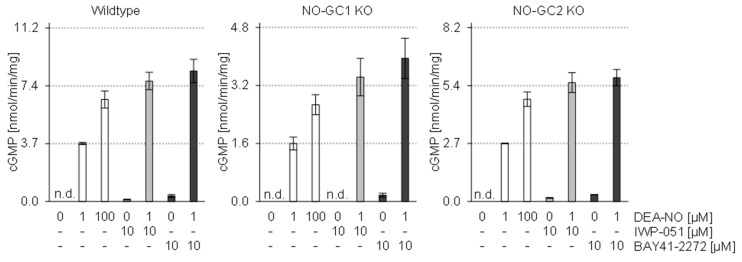
In vitro assays with brain homogenates do not show NO-GC1-specific activity of IWP-051. cGMP-forming activities of whole brain homogenates of wildtype, NO-GC1 KO and NO-GC2 KO mice were measured by [^32^P] cGMP formation. Homogenates were incubated with the indicated concentrations of DEA/NO, IWP-051, and Bay 41-2272. As the knockout of either one of the NO-GC isoforms reduces the cGMP-forming activity, the panels have different ordinates. Open, light gray and dark gray bars show activities in the absence of NO-GC stimulators, in the presence of IWP-051 and in the presence of BAY41-2272, respectively. Data are shown as mean ± SD of three independent biological replicates analyzed for each genotype (*n* = 9 mice in total); n.d., not detectable.

The potential preference of IWP-051 for stimulation of NO-GC1 vs. NO-GC2 was further evaluated in vitro. The cGMP-forming activity of brain homogenates of wildtype, NO-GC1 KO and NO-GC2 KO mice was determined by measuring [^32^P] cGMP formation (Figure 7). Whole brain homogenates were incubated with DEA/NO (1 µM, 100 µM), IWP-051 (10 µM) or Bay 41-2272 (10 µM) or a combination of a sub-maximally effective concentration of DEA/NO (1 µM) and one of the NO-GC stimulators. Interestingly, both compounds potentiated NO-induced cGMP formation in wildtype, NO-GC1 KO and NO-GC2 KO homogenates indicating that IWP-051 and Bay 41-2272 had a similar activity profile and stimulated both NO-GC1 and NO-GC2 under the assay conditions.

## 3. Discussion

The optimization of established therapies as well as the development of new drugs is important to improve the treatment of diseases with poor prognoses. In the past decades, several drugs were developed that affect the cGMP signaling cascade, such as PDE inhibitors [39,40] and NO-GC stimulators [41,42]. These drugs are mainly applied to treat cardiovascular diseases. However, the development of cGMP-based therapies for disorders of the CNS is lagging behind, in part because the influence of these drugs on cGMP levels in distinct brain regions are not completely resolved. In the present study, we analyzed the cGMP-elevating effects of NO and two NO-GC stimulators, IWP-051 and Bay 41-2272, in various brain regions by real-time imaging of cGMP in live acute brain slices of cGMP sensor mice.

Application of NO to our brain slices resulted in robust cGMP increases in Purkinje cells and granule neurons of the cerebellum, the striatum and the hippocampal CA1 area. These results are in line with previous work showing the presence of the NO-cGMP signaling pathway in these brain regions [35,43,44,45,46,47]. IWP-051 potentiated NO-induced cGMP signals in cerebellar Purkinje cells and granule neurons as well as in the striatum, but, surprisingly, failed to potentiate NO-induced cGMP signals in the hippocampal CA1 area. Control experiments with the NO-GC blocker ODQ and the PDE inhibitor IBMX showed that basal levels of cGMP production via NO-GC and degradation via PDEs were low in our hippocampal slices. Importantly, IBMX further increased NO-induced cGMP levels, thus, excluding the possibility that the failure to detect cGMP potentiation by IWP-051 was due to already maximal cGMP levels or saturation of the cGMP sensor after stimulation with NO alone. Consistent with brain slice imaging data, IWP-051 potentiated NO-induced cGMP signals in primary CGNs, but not in primary HNs. In contrast to IWP-051, Bay 41-2272 enhanced NO-induced cGMP in both hippocampal slices and primary neurons. The finding that Bay 41-2272 was active in the hippocampus makes it unlikely that a potential potentiating effect of IWP-051 was not detected because NO alone had stimulated hippocampal NO-GC already to its maximal activity. Interestingly, mRNA expression data of NO-GC1 and NO-GC2 correlated with the differential activity profile of IWP-051 in the cerebellum, striatum, and hippocampus and pointed to preferential stimulation of NO-GC1 by IWP-051.

Based on these results, we hypothesized that IWP-051 is a NO-GC1-specific stimulator, in contrast to Bay 41-2272, which acts on both NO-GC1 and NO-GC2. The hypothesis was tested by comparative cGMP imaging of acute brain slices from wildtype, NO-GC1 KO and NO-GC2 KO mice. Indeed, IWP-051 potentiated NO-induced cGMP signals in the striatum of wildtype and NO-GC2 KO mice, but was inactive in the striatum of NO-GC1 KO mice. Bay 41-2272 was active in striatal brain slices from mice of all genotypes. Considering that the striatum expresses NO-GC1 and NO-GC2, we conclude that the cGMP-enhancing activity of IWP-051 in acute brain slices requires NO-GC1 but not NO-GC2, while Bay-41-2272 stimulates both NO-GC1 and NO-GC2. 

The differential ability of IWP-051 to enhance cGMP in different brain regions could be due to differential penetration of the compound into different regions of our acute brain slices, with good penetration into the cerebellum and striatum and inefficient penetration into the hippocampal CA1 region. It is, however, very unlikely that the lack of activity of IWP-051 in hippocampal slices was indeed due to a penetration problem because the compound was also inactive in primary cultures of hippocampal neurons, which should not pose a penetration barrier. Thus, the simplest explanation for the different ability of IWP-051 and Bay 41-2272 to potentiate NO-induced cGMP in different brain areas would be that IWP-051 stimulates preferentially NO-GC1, while Bay 41-2272 acts on both NO-GC isoforms. Since the hippocampal CA1 area expresses predominantly NO-GC2, the Bay compound but not the IWP compound was active in this brain region. In addition to stimulation of NO-GC, part of the cGMP-enhancing effects of Bay 41-2272 [20,21] and IWP-051 [28] may be attributable to inhibition of PDE5. In our imaging experiments, it is difficult to distinguish cGMP generated from activation of NO-GC vs. inhibition of PDEs. Thus, the differential activity of IWP-051 and Bay 41-2272 in different brain regions could be related to the presence of different NO-GC isoforms and/or different PDE isoforms in different neurons.

It is not clear how NO-GC1 isoform specificity of IWP-051 could be achieved at the molecular level, given that NO-GC1 and NO-GC2 are highly similar proteins [48] and Bay 41-2272 and IWP-051 were both described to stimulate NO-GC in a heme-dependent manner [19,28]. Indeed, to our knowledge, isoform-specific NO-GC modulators have not been reported to date. Interestingly, NO-GC1 is predominantly localized in the cytosol, whereas NO-GC2 is membrane-associated [49]. One possibility to explain our finding that IWP-051 preferentially stimulates NO-GC1, while Bay 41-2272 stimulates both NO-GC1 and NO-GC2, could be that these compounds show different subcellular partitioning based on their different physicochemical properties. In contrast to Bay 41-2272, the distribution of the IWP compound to membrane compartments, where NO-GC2 is localized, may be limited thus resulting in preferential stimulation of NO-GC1 vs. NO-GC2 in brain slices and primary neurons.

It is important to note that our in vitro measurements of the cGMP-forming activities of whole brain homogenates prepared from wildtype, NO-GC1 KO and NO-GC2 KO mice showed a similar activity profile of IWP-051 and Bay 41-2272 for both NO-GC isoforms with no evidence for NO-GC1-specific activity of IWP-051. At present, we can only speculate about the reason(s) behind the discrepancy between cGMP data regarding NO-GC-specific effects of IWP-051 obtained with brain slices and primary neurons vs. brain homogenates. Clearly, the methods and conditions used for cGMP measurements in this study differed in several important aspects. FRET/cGMP imaging with our biosensor mice allows the measurement of cGMP levels in intact cells of selected brain regions and, in the case of brain slices, in a largely preserved tissue architecture, while the in vitro cGMP assay was performed with homogenates obtained from whole brains. The concentrations of IWP-051 and Bay 41-2272 used for stimulation of brain homogenates were the same as the highest concentrations used for intact cells in our brain slice studies (10 µM). However, it is likely that NO-GC in homogenates was exposed to higher effective concentrations of IWP-051 and Bay 41-2272 than NO-GC in brain slices. The isoform-specificity of the IWP compound might have been lost in brain homogenates under these conditions. Another difference was that the cGMP measured in brain slices of neuron/glia-specific cGMP sensor mice was derived from neuronal and glial cells, while the cGMP-forming activity determined in brain homogenates was also derived from cerebral blood vessels, which express both NO-GC isoforms and generate high amounts of cGMP in response to NO. Based on the cGMP imaging data one would have expected that IWP-051 does not potentiate NO-induced cGMP-forming activity in brain homogenates of NO-GC1 KO mice. However, the contrary was observed. Obviously, the concentration of 10 µM IWP-051 has resulted in considerable activation of NO-GC2 in homogenates of NO-GC1 KO brains. In the future, the in vitro cGMP assay should be optimized, so that one can analyze individual brain regions (e.g., striatum vs. hippocampus). On the other hand, we cannot exclude the possibility that the apparent isoform-selectivity of IWP-051 observed by FRET-based cGMP imaging of acute brain slices and primary neurons was due to presently unknown limitations of this relatively new technique.

In sum, FRET-based cGMP imaging in acute brain slices revealed that the NO-GC stimulators IWP-051 and Bay 41-2272 potentiate NO-induced cGMP increases in several brain areas. Interestingly, IWP-051 was active in the cerebellum and striatum, but, in contrast to Bay 41-2272, not in the hippocampus. mRNA expression data and genetic evidence from knockout models suggest that the differential activity profile of IWP-051 is due to preferential stimulation of NO-GC1 vs. NO-GC2. It is tempting to speculate that IWP-051 might be the first NO-GC modulator acting in an isoform-specific manner, at least in acute brain slices. However, no preference of the IWP compound for NO-GC1 was observed when whole brain homogenates were analyzed indicating that the methods and conditions of cGMP measurements can affect results with NO-GC stimulators. These results call for further experiments testing the activity of IWP-051 or CNS-penetrant derivatives thereof on brain functions in vivo. In particular, it would be interesting to compare IWP’s effects on cerebellar and striatal functions (e.g., motor learning and reward systems) vs. hippocampal functions (e.g., spatial learning). This might be a starting point to develop novel brain region-specific drugs for disorders of the nervous system.

## 4. Materials and Methods

### 4.1. Mice

All animal procedures were approved by the Regierungspräsidium Tübingen (IB 1/15, 21 August 2015) and performed in compliance with the humane care and use of laboratory animals. Mice were housed at 22 °C and 50–60% humidity in a 12 h light/12 h dark cycle with access to standard rodent chow and tap water. The following mouse lines were used: R26-CAG-cGi500(L1) [31], R26-CAG-cGi500(L2) [31], L7Cre [50], NesCre [51], NO-GC1 KO [52], and NO-GC2 KO [52]. R26-CAG-cGi500(L1) were directly used for experiments. Other experimental animals were generated by crossing mouse lines to obtain the following cross mice: L7Cre;R26-CAG-cGi500(L2), NesCre;R26-CAG-cGi500(L2), NesCre;R26-CAG-cGi500(L2);NO-GC1 KO, and NesCre;R26-CAG-cGi500(L2);NO-GC2 KO. If not stated otherwise, experiments were performed with 2- to 12-month-old mice of either sex on a mixed 129Sv/C57BL6N genetic background. Genotyping of the animals was performed by PCR analysis of ear puncture DNA using the following primers: for detection of the R26-CAG-cGi500(L1) and R26-CAG-cGi500(L2) allele, BB01 (CTCTGCTGCCTCCTGGCTTCT), BB02 (CGAGGCGGATCACAAGCAATA), and BB03 (TCAATGGGCGGGGGTCGTT), which amplify a 330-bp fragment of the wildtype allele (BB01 and BB02) and a 250-bp fragment of the transgene (BB01 and BB03); for NesCre and L7Cre transgenes, Cre800 (GCTGCCACGACCAAGTGACAGCAATG) and Cre1200 (GTAGTTATTCGGATCATCAGCTACAC), which amplify a 402-bp fragment of the Cre transgene; for NO-GC1 KO mice, BB12 (ATGACAAATGAGCAGACG), BB13 (TCCCGAGATGAAGTAGTTAGT), and BB14 (TGTAGAAGAGGGGATAGAAAGACC), which amplify an 879-bp fragment of the wildtype allele (BB12 and BB13) and a 997-bp fragment of the knockout allele (BB13 and BB14); for NO-GC2 KO mice, BB15 (TTTGAAATTACTTGGAGATAGA), BB16 (AGGTGGGGCTGTCTCTGAA), BB17 (AGGTGGGGCTGTCTCTGAA), and BB18 (GGGGGCCCTGACATTTGA), which amplify a 760-bp fragment of the wildtype allele (BB15 and BB16) and a 513-bp fragment of the knockout allele (BB17 and BB18).

### 4.2. Drugs

The following drugs were used: ANP and CNP (Tocris, Minneapolis, MN, USA), Bay 41-2272 (Santa Cruz Biotechnology, Dallas, TX, USA), DEA/NO (Axxora, Ann Arbor, MI, USA), IBMX (Sigma, St. Louis, MO, USA), IWP-051 (Ironwood Pharmaceuticals, Cambridge, MA, USA), and ODQ (Axxora).

### 4.3. Cell Culture 

To establish primary cultures of cerebellar granule neurons (CGNs), the following solutions and “tubes” were prepared: 10× Krebs buffer (1.24 M NaCl, 54 mM KCl, 5 mM NaH_2_PO_4_, pH 7.4); 0.3% bovine serum albumin (BSA, Roth, Karlsruhe, Germany) solution in 1× Krebs buffer, 14.3 mM D-glucose, 2.5 mM MgSO_4_, sterilized by filtration; “tube 1” contained 30 mL 0.3% BSA solution; “tube 2” contained 30 mL 0.3% BSA solution and 300 μL trypsin solution [2.5× trypsin-EDTA (Thermo Scientific, Waltham, MA, USA) in PBS], added shortly before use; “tube 3” contained 7.8 mg trypsin inhibitor (Life Technologies, Carlsbad, CA, USA) in 15 mL 0.3% BSA solution, 3.1 mM MgSO_4_, and 0.1 mg/mL DNase (Roche, Basel, Switzerland), added shortly before use); in “tube 4”, 17 mL 0.3% BSA solution were mixed with 8 mL solution from “tube 3” and then 10 mL were discarded, so that 15 mL remained in “tube 4”; “tube 5” contained 12.5 mL 0.3% BSA solution, 2.5 mM MgSO_4_, and 0.1 mM CaCl_2_. Two to five cerebella from 7-day-old mice were isolated and the surrounding meninges were removed in 0.3 % BSA solution. Then, the cerebella were homogenized in 2 mL 0.3% BSA solution with a pipette, transferred into “tube 1” and centrifuged at 170 g for 5 min at room temperature. The supernatant was removed and the pellet was resuspended in the solution from “tube 2” and incubated for 15 min at 37 °C with gentle shaking every 5 min. Then, the suspension was transferred into “tube 4”, mixed and centrifuged at 170 g for 5 min at room temperature. The supernatant was removed and the cell pellet was resuspended in the solution from “tube 3” by pipetting 10 times with a Pasteur pipette. Then, the suspension was transferred into “tube 5”, mixed, passed through a netwell mesh (70 µm), and centrifuged at 170 g for 5 min at room temperature. The pellet was resuspended in CGN medium [Minimal Essential Medium (Life Technologies) containing 22 mM KCl, 2% B27 supplement (Thermo Scientific), 9% fetal bovine serum (Thermo Scientific), 0.3 mM glutamine (Thermo Scientific), and 0.1 mM gentamicin (Thermo Scientific)] and plated on 24-well plates (100 k cells per well, viability >95% as determined by trypan blue staining). Wells were equipped with glass coverslips, which have been coated overnight with poly-D-lysine (20 µg/mL, Thermo Scientific). CGNs were grown at 37 °C and 5% CO_2_ and after 24 h cytosine arabinoside (5 µM, Sigma) was added. Every 3 days half of the medium was changed.

Primary cultures of hippocampal neurons (HNs) were prepared from mice of postnatal day 0. Brains were dissected and hippocampi separated from remaining brain. For short-term storage of hippocampi for up to 1 h, Ca^2+^/Mg^2+^-free Hank’s Balanced Salt Solution (1×, Life Technologies) supplemented with HEPES (10 mM, pH 7.3), was used. After removal of meninges and blood vessels, the tissue was trypsinized in 2 mL Hank’s Balanced Salt Solution (1×) supplemented with HEPES (10 mM, pH 7.3) and 40 µL trypsin solution (2.5× trypsin-EDTA in PBS) for 15 min at 37 °C. Then, 0.1 µg/mL DNase (Roche) was added, the suspension was incubated for 1 min at room temperature, and 10 mL Dulbecco’s Modified Eagle’s Medium (DMEM, Life Technologies) supplemented with 10% horse serum (Life Technologies) were added. The suspension was incubated for ~30 s until tissue pieces were settled down. Then, the supernatant was carefully removed with a pipette and 10 mL DMEM supplemented with 10% horse serum were added. The suspension was incubated for ~30 s until tissue pieces were settled down and the supernatant was removed as described above. Then, tissue pieces were dissociated by pipetting up and down in 2 mL DMEM supplemented with 10% horse serum. After centrifugation at 50 g for 5 min at room temperature, the pellet was resuspended in HN medium (Minimal Essential Medium containing 1 mM Na pyruvate, 0.6% d-glucose, 2 mM L-glutamine, and 2% B27 supplement). Cells were plated in 24-well plates on poly-d-lysine-coated glass coverslips (200–400 k cells per well, viability >95% as determined by trypan blue staining) and grown at 37 °C and 6% CO_2_. Cytosine arabinoside (5 µM, Sigma) was added 24 h after plating.

### 4.4. Preparation of Acute Brain Slices and Fixed Brain Sections 

For the preparation of acute brain slices, brains were dissected and cut in ice-cold carbogen-gassed Ringer buffer (126 mM NaCl, 2.5 mM KCl, 1 mM MgCl_2_, 1 mM CaCl_2_, 1.25 mM NaH_2_PO_4_, 26 mM NaHCO_3_, 20 mM D-glucose). Slicing was performed with a vibratome (VT1200, Leica, Buffalo Grove, IL, USA). Brains were cut sagittally into 300-µm slices. Then, slices were incubated in 37 °C pre-warmed carbogen-gassed Ringer buffer and imaging experiments were performed 1 h after slicing at room temperature.

For the preparation of fixed brain sections, brains of 8- to 12-week-old mice were incubated in 4% ice-cold paraformaldehyde (Roth) for 5 h at 4 °C, washed with PBS and stored in 30% sucrose (Roth) in PBS for 1–2 days at 4 °C until brains were sunk down. Then, brains were embedded in Tissue Tek O.C.T. compound (Sakura Finetek Germany GmbH, Staufen, Germany) and 10-µm sections were prepared using a cryotome (Microm, Thermo Fisher, Waltham, MA, USA) and mounted on SuperFrost glass slides (Thermo Fisher). Sections were incubated in PBS for 5 min, then in PBS containing 0.4% Triton X-100 (Roth) for 5 min, and then cell nuclei were stained with 1 µg/mL Hoechst 33258 (Sigma) in PBS containing 0.4% Triton X-100 for 10 min at room temperature.

### 4.5. Imaging

FRET/cGMP imaging of cultured neurons was performed 5–7 days after plating using an epifluorescence setup as described previously [31,33,53]. Briefly, the setup consisted of an inverted Axiovert 200 microscope (Zeiss, Oberkochen, Germany) equipped with a NeoFluar40×/1.30 oil objective, a light source with excitation filter switching device (Oligochrome, TILL Photonics GmbH, Graefelfing, Germany), a Dual-View beam splitter (Photometrics, Tucson, AZ, USA) with 516 nm dichroic mirror and emission filters for CFP (480/30 nm) and YFP (535/40 nm), and a charge-coupled device camera (Retiga 2000R; QImaging, Surrey, BC, Canada). The cells were superfused at room temperature at a flow rate of 1 mL/min with imaging buffer (140 mM NaCl, 5 mM KCl, 1.2 mM MgSO_4_, 2.5 mM CaCl_2_, 5 mM D-glucose, 5 mM HEPES, pH 7.4) or imaging buffer supplemented with drugs. Drugs were applied via sample loops for 2 min corresponding to a volume of 2 mL. To remove the buffer from the system, a vacuum pump with adjustable vacuum (Laboport N86, KNF Neuberger, Freiburg, Germany) was connected to the system [31,33].

For FRET/cGMP imaging of acute brain slices, a spinning disk imaging system was used. The setup consisted of an upright Examiner.Z1 microscope (Zeiss), a Yokogawa CSU-X1 spinning disk confocal scanner, three diode lasers (445 nm, 488 nm and 561 nm), three water immersion objectives [W N-ACHROMAT 10×/0.3, W Plan-APOCHROMAT 20×/1.0 DIC (UV) VIS-IR, W Plan-APOCHROMAT 40×/1.0 DIC VIS-IR; all from Zeiss] and one air objective [EC Plan-NEOFLUAR 2.5×/0.085; Zeiss]. For FRET-based imaging, the donor fluorophore CFP was excited with the 445 nm laser, and a Dual-View beam splitter (Photometrics) with 505 nm dichroic mirror, 470/24 nm and 535/30 nm emission filters was used for simultaneous acquisition of CFP and YFP. Signals were recorded with an electron-multiplying charged-coupled device (EM-CCD) camera (QuantEM 512SC, Photometrics) at a frame rate of 0.2 Hz and an exposure time of 300 ms. The system was controlled by VisiView software (Visitron Systems, Puchheim, Germany). A CoolLED pE-2 LED system was used for epifluorescence illumination at 400 nm, 450 nm, 500 nm and 561 nm. During real-time imaging, the tissue was continuously superfused with carbogen-gassed Ringer buffer or buffer containing drugs of interest at a flow rate of 1 mL/min at room temperature. A custom-built superfusion system was used consisting of a FPLC pump (Pharmacia P-500, GE Healthcare, Chicago, IL, USA), FPLC injection valves (Pharmacia V-7, GE Healthcare), a magnetic platform (Warner Instruments, Hamden, CT, USA), a superfusion chamber (RC-26, Warner Instruments), a Slice Hold-Down (SHD-26H/10, Warner Instruments) and sample loops of different sizes for application of drugs. To ensure that drug exposure was comparable between different drugs and tissue slices, slices were perfused for the same time span with the same volume of drug solution. To remove the buffer from the system, a vacuum pump with adjustable vacuum (Laboport N86, KNF Neuberger) was connected to the system [31,33].

### 4.6. Data Base Research

In situ hybridization data of NO-GC1 and NO-GC2 expression were taken from the Allen mouse brain atlas [36]. Sagittal brain sections of a C57BL/6 mouse were analyzed for the genes *Gucy1a3*, *Gucy1a2*, and *Gucy1b3* encoding the NO-GC α_1_ subunit, α_2_ subunit, and β_1_ subunit, respectively.

### 4.7. Determination of cGMP-Forming Activities

Whole mouse brains were homogenized in 5 volumes of 50 mM NaCl, 50 mM triethanolamine/HCl, pH 7.4, 1 mM EDTA, protease inhibitor cocktail (1:100, Sigma) using a glass/teflon homogenizer. Debris was removed by centrifugation (15 min, 21,000× *g*, 4 °C) and protein content was determined by Bradford protein assay (BIO-RAD). cGMP-forming activity was determined by conversion of [α-^32^P] GTP as described before [54]. In short, 40–50 µg of protein were incubated for 10 min with 500 µM [α-^32^P] GTP (≈3 kBq), 3 mM MgCl_2_, 1 mM cGMP, 3 mM dithiothreitol (DTT), 0.5 mg/mL BSA, 0.025 mg creatine phosphokinase/sample, 5 mM creatine phosphate, and 1 mM IBMX in 50 mM triethanolamine/HCl, pH 7.4, in the presence of 1 or 100 µM DEA-NO, 10 µM IWP-51 and 10 µM Bay 41-2272 as indicated. Each homogenate was measured in triplicates. Reactions were stopped and formed cGMP was determined as described [54].

### 4.8. Analysis and Statistics

For image acquisition and online analysis, VisiView (Visitron) was used, and for offline analysis Fiji software (NIH) [55]. For further analysis, Microsoft Excel (Microsoft) and Origin (OriginLab) were used. F_480_ signals (CFP emission, cyan traces in respective graphs) and F_535_ signals (YFP emission, yellow traces in respective graphs) were background-corrected and then used to calculate the F_480_/F_535_ ratio R (black traces in respective graphs). ΔF_480_/F_480_, ΔF_535_/F_535_, and ΔR/R traces were obtained by normalization to the baseline recorded for ~3 min at the beginning of each experiment. For ΔR/R peak area and peak height calculation, Peak Area/Height Analyzer of Origin was used. Peak borders were defined manually. Statistical analysis was performed using Origin software. Statistical differences between more than two groups were analyzed by one-way ANOVA followed by Bonferroni’s test. *p* values < 0.05 were considered to be significant.

## Abbreviations

ANP	Atrial natriuretic peptide
BSA	Bovine serum albumin
CAG	CMV immediate early enhancer, chicken β-actin and rabbit β-globin
CFP	Cyan fluorescent protein
cGi	cGMP indicator
cGMP	Cyclic 3’ 5’ guanosine monophosphate
CGN	Cerebellar granule neuron
CNP	C-type natriuretic peptide
DEA/NO	2-(N,N-diethylamino)-diazenolate-2-oxide diethylammonium salt
DMEM	Dulbecco’s modified Eagle medium
FRET	Förster/Fluorescence resonance energy transfer
GCL	granule cell layer
HN	Hippocampal neuron
IBMX	3-isobutyl-1-methylxanthin
NO	Nitric oxide
NO-GC	NO-sensitive guanylyl cyclase
ODQ	1H-[1,2,4]oxadiazolo[4 ,3-a]quinoxalin-1-one
PBS	Phosphate buffered saline
PDE	Phosphodiesterase
ROI	Region of interest
YFP	Yellow fluorescent protein
